# Genetic Testing for *TMEM154* Mutations Associated with Lentivirus Susceptibility in Sheep

**DOI:** 10.1371/journal.pone.0055490

**Published:** 2013-02-11

**Authors:** Michael P. Heaton, Theodore S. Kalbfleisch, Dustin T. Petrik, Barry Simpson, James W. Kijas, Michael L. Clawson, Carol G. Chitko-McKown, Gregory P. Harhay, Kreg A. Leymaster, the International Sheep Genomics Consortium

**Affiliations:** 1 U.S. Meat Animal Research Center (USMARC), Clay Center, Nebraska, United States of America; 2 Department of Biochemistry and Molecular Biology, School of Medicine, University of Louisville, Louisville, Kentucky, United States of America; 3 Intrepid Bioinformatics, Louisville, Kentucky, United States of America; 4 GeneSeek, a Neogen company, Lincoln, Nebraska, United States of America; 5 Division of Animal, Food and Health Sciences, CSIRO, Brisbane, Australia; CNRS UMR7275, France

## Abstract

In sheep, small ruminant lentiviruses cause an incurable, progressive, lymphoproliferative disease that affects millions of animals worldwide. Known as ovine progressive pneumonia virus (OPPV) in the U.S., and Visna/Maedi virus (VMV) elsewhere, these viruses reduce an animal’s health, productivity, and lifespan. Genetic variation in the ovine transmembrane protein 154 gene (*TMEM154*) has been previously associated with OPPV infection in U.S. sheep. Sheep with the ancestral *TMEM154* haplotype encoding glutamate (E) at position 35, and either form of an N70I variant, were highly-susceptible compared to sheep homozygous for the K35 missense mutation. Our current overall aim was to characterize *TMEM154* in sheep from around the world to develop an efficient genetic test for reduced susceptibility. The average frequency of *TMEM154* E35 among 74 breeds was 0.51 and indicated that highly-susceptible alleles were present in most breeds around the world. Analysis of whole genome sequences from an international panel of 75 sheep revealed more than 1,300 previously unreported polymorphisms in a 62 kb region containing *TMEM154* and confirmed that the most susceptible haplotypes were distributed worldwide. Novel missense mutations were discovered in the signal peptide (A13V) and the extracellular domains (E31Q, I74F, and I102T) of *TMEM154*. A matrix-assisted laser desorption/ionization–time-of flight mass spectrometry (MALDI-TOF MS) assay was developed to detect these and six previously reported missense and two deletion mutations in *TMEM154*. In blinded trials, the call rate for the eight most common coding polymorphisms was 99.4% for 499 sheep tested and 96.0% of the animals were assigned paired *TMEM154* haplotypes (i.e., diplotypes). The widespread distribution of highly-susceptible *TMEM154* alleles suggests that genetic testing and selection may improve the health and productivity of infected flocks.

## Introduction

Visna/Maedi virus (VMV) and its closely related North American counterpart, ovine progressive pneumonia virus (OPPV), are small ruminant lentiviruses (SRLV) of the retroviridae family that infect sheep around the world (for review see [1]). Infections are life-long and there are no effective treatments or vaccines [2]. The first signs of disease typically appear after age two and often include the loss of body condition and indurative mastitis (hard udder). Disease progression is associated with severe clinical signs that include difficulty breathing, chronic wasting, loss of motor control, and arthritis. Significant transmission occurs horizontally among adult sheep by respiratory routes, and vertically between dam and offspring by ingestion of infected colostrum [3], [4].

The impact of SRLV infection is considerable when the virus is introduced into a naïve flock with susceptible sheep. The mortality in such flocks may reach 30% per year after a few years [5]. Once established in a ewe flock, subclinical infection weakens the resistance to disease, decreases fertility, and reduces lamb production [6]. In the U.S., a quarter of the sheep are infected with OPPV and a third of sheep operations test positive [7]. Infected ewes have difficulty raising lambs and also transmit infection to their offspring and other flockmates. Collectively, complications of SRLV infections lead to animal pain, disability, early culling, and increased labor.

Stepwise strategies for SRLV disease control typically begin with the removal of infected sheep and focus on lowering the infection prevalence [4]. SRLV can also be eradicated in one production cycle by isolating all neonates from their infected dams, raising the lambs on uninfected colostrum and milk, and maintaining them separately from infected animals thereafter. Although these methods have been successful in eradicating SRLV in sheep, they require a significant commitment of time and resources, and flocks remain vulnerable to SRLV outbreaks if exposed to infected animals [8]. Efforts to eradicate SRLV in sheep and maintain an infection-free status would be enhanced by the use of replacement breeding stock that are genetically resistant to lentivirus infections.

The discovery of ovine transmembrane protein gene 154 (*TMEM154*) as a major OPPV susceptibility gene [9] provides an opportunity to produce sheep that are less susceptible to infection. The function of the TMEM154 protein has not yet been reported for any species and remains unknown. However, the ancestral *TMEM154* haplotype in sheep is common and predicted to encode a precursor protein of 191 amino acids that is cleaved to a mature protein with 161 residues. Two other haplotypes encoding polypeptide isoforms are also common in U.S. sheep and, together with the ancestral haplotype, account for more than 97% of those observed [9]. The ancestral *TMEM154* haplotype (designated haplotype 3, GenBank accession JX961707) encodes glutamate (E) at position 35 and asparagine (N) at position 70. Haplotype 2 has an isoleucine (I) mutation at position 70 while haplotype 1 has a lysine (K) mutation at position 35. A case-control study with 130 pairs of 4- to 9-year old ewes matched for lifetime exposure showed the odds of being infected were 69 times greater for those with one copy of either haplotype 2 or 3, compared to those homozygous for haplotype 1 (*p*-value <0.0001, 95% CI 12–2800) [9]. Likewise, a cohort study involving 2705 unmatched U.S. sheep from Nebraska, Idaho, and Iowa showed the relative risk of infection was 2.85 times greater for sheep with one copy of either haplotype 2 or 3, compared to those homozygous for haplotype 1 (*p*-value <0.0001, 95% CI 2.36–3.43) [9]. Also reported were two naturally occurring *TMEM154* deletion mutations, R4A(delta53) and E82Y(delta82), that were predicted to abolish the protein’s function. Although rare, sheep homozygous for R4A(delta53) remained healthy, productive, and uninfected despite a long lifetime of significant exposure. In addition to its association with susceptibility to OPPV infection, *TMEM154* was also recently reported to be associated with the abundance of integrated provirus (i.e. viral load) in Rambouillet, Polypay, and Columbia sheep in the U.S. [10]. Taken together, these observations suggest that removing sheep with the most susceptible *TMEM154* alleles may help eradicate OPPV and protect flocks from reinfection.

While these findings show promise for improving U.S. sheep populations, it is important to identify which other populations around the world may be impacted by highly-susceptible *TMEM154* haplotypes. Estimating a population’s relative vulnerability to disease helps establish guidelines for preventative measures before an outbreak occurs. Previous research in U.S. sheep showed [9] the combined frequency of the highly-susceptible *TMEM154* haplotypes could be estimated with a DNA marker on the OvineSNP50 BeadChip. This was possible because the “c” nucleotide allele of the single nucleotide polymorphism (SNP) OAR17_5388531 on the OvineSNP50 BeadChip is in strong linkage disequilibrium (LD) with the E35 allele located approximately 10 kb upstream (*r* ^2^ = 0.98). The major E35-containing haplotypes include the ancestral form of *TMEM154* (haplotype 3) and the I70 variant (haplotype 2). Together, these two alleles accounted for more than 91% of the alleles containing E35 in U.S. sheep [9]. Thus, the frequency of the ‘c’ allele of OAR17_5388531 was a reasonable estimate of genetic predisposition to OPPV infection in U.S. sheep. The overall aim of the present research was to characterize *TMEM154* in sheep from around the world to develop an efficient genetic test for reduced susceptibility. The findings indicated that most sheep populations around the world have highly-susceptible forms of *TMEM154* and the genetic test described here efficiently detected eight known variant forms and four novel *TMEM154* haplotypes discovered during this project. This genetic test, and similar designs adapted to other genotyping platforms, may be useful for preventing or controlling ovine lentivirus infections through selective breeding.

## Results

### LD between *TMEM154* E35K and OAR17_5388531 in an International Panel of 75 Sheep

The availability of next-generation whole genome sequence data from the International Sheep Genomics Consortium (ISGC) provided the opportunity to directly determine *TMEM154* genotypes *in silico* for 75 sheep sampled from 39 breeds and two wild species from around the world (45 groups in total). The data set for each animal represented approximately 10-fold genome coverage and was used to estimate LD between the SNP alleles for *TMEM154* E35K and those for OAR17_5388531. In this set of 75 sheep, the *r* ^2^ statistic for these two markers was 0.87 and thus indicated relatively strong LD. Only four sheep from three breeds had alleles that were unambiguously in the opposite phase (i.e., the E35 allele was linked to the ‘t’ allele of OAR17_5388531). The high *r* ^2^ statistic indicated that SNP OAR17_5388531 could provide a reasonable estimate of highly-susceptible *TMEM154* alleles in most sheep populations.

### Estimating the Frequency of the Highly-susceptible *TMEM154* Alleles in Sheep Breeds from Around the World

**Figure 1:**
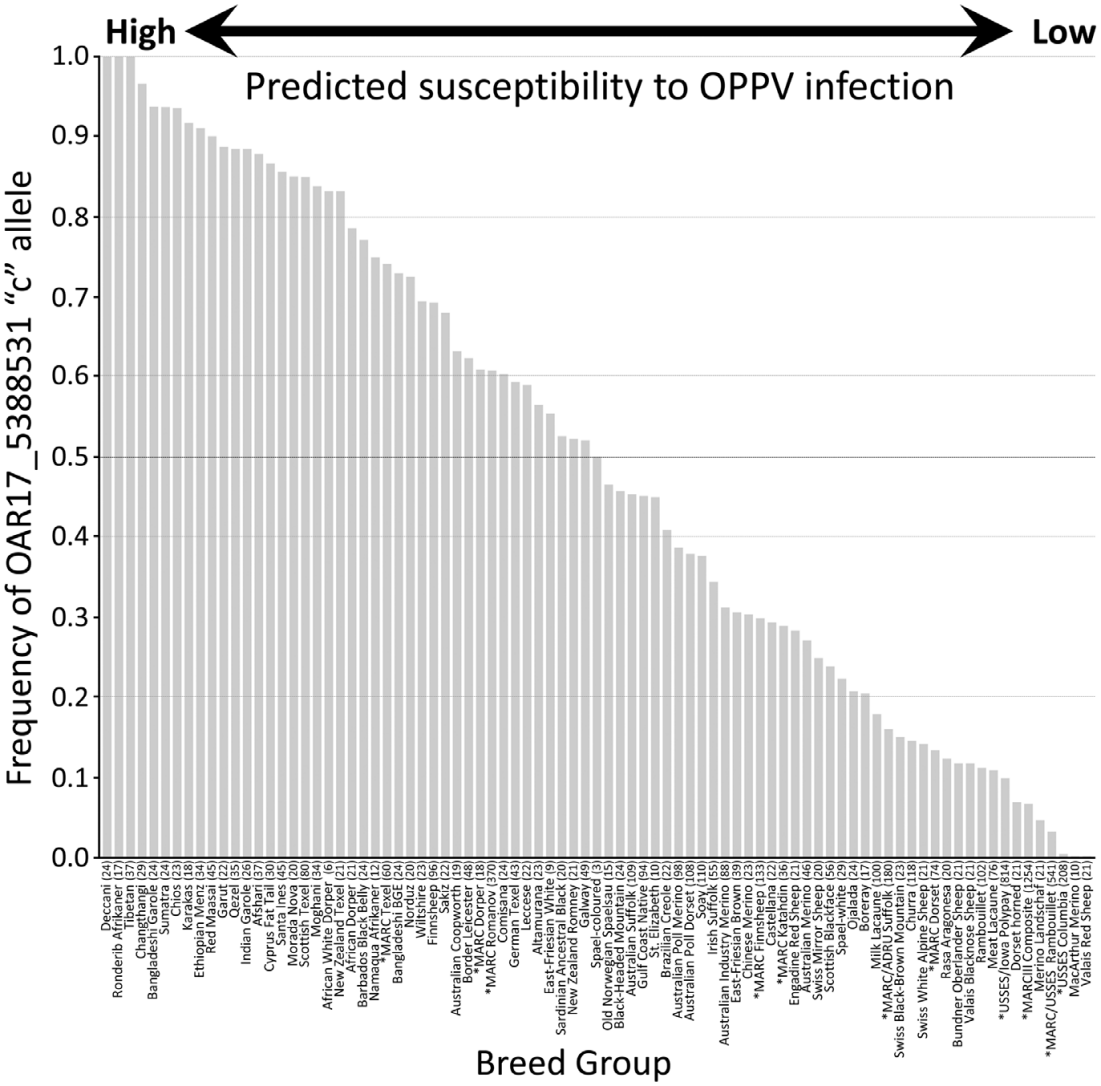
Estimating the frequency of highly-susceptible *TMEM154* alleles in global sheep populations. The “c” allele of SNP OAR17_5388531 is in linkage disequilibrium with the “g” nucleotide allele in codon 35 (gaa) of *TMEM154*. Genotypes for OAR17_5388531 were derived from the ISGC ovine SNP50k data set [11]. Numbers in parentheses for each breed group indicate the number of animals genotyped. The 11 breed groups with asterisks were genotyped for *TMEM154* E35 by Sanger sequencing [9] and were included for comparison with the 74 ISGC breed groups.

In a collection of 2,759 sheep DNAs from 74 breeds from around the world, the frequency of the “c” nucleotide allele of the c/t SNP OAR17_5388531 ranged from 0.0 to 1.0 with a mean of 0.51 and median of 0.50 among breeds (Figure 1). Breed groups with the highest “c” allele frequencies are predicted to have high frequencies of *TMEM154* E35 (haplotypes 2 and 3) and larger proportions of highly-susceptible animals. Conversely, breed groups with low “c” allele frequencies are predicted to have more homozygous K35 animals (haplotype 1) and be less susceptible to OPPV. The results indicated that the highly-susceptible *TMEM154* alleles are present in breeds throughout the world.

### Discovering Novel *TMEM154* SNPs and Missense Mutations in an International Panel of 75 Sheep

**Figure 2:**
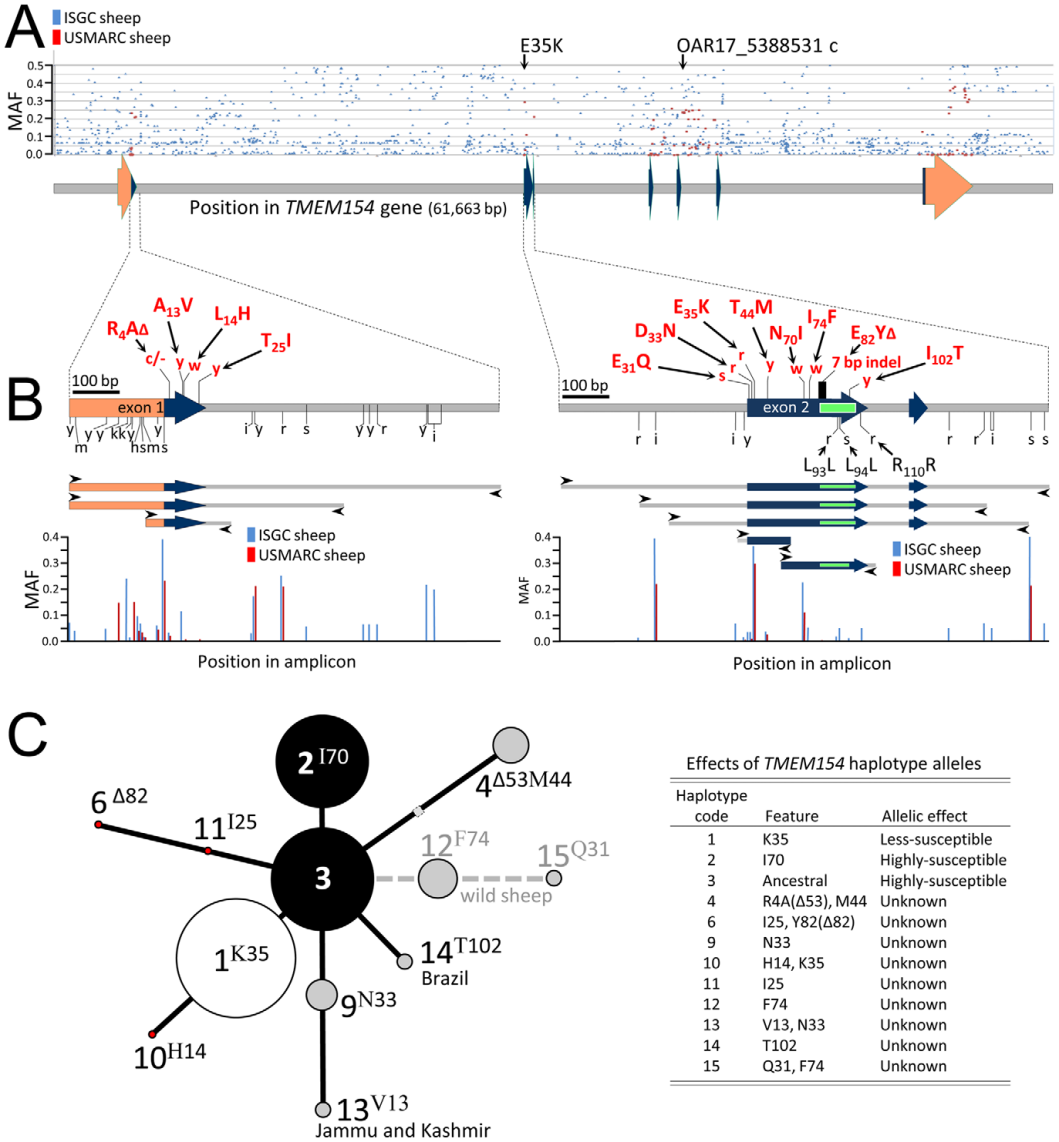
*TMEM154* SNP maps and median-joining networks. Panel A, genomic map of *TMEM154*: orange arrows, 5′ and 3′untranslated regions of exons; blue arrows, exon coding regions; grey rectangles, introns or intergenic regions. Blue and red tick dots denote position and frequency of SNPs in an international panel of 75 sheep and a panel of 96 U.S. sheep [9], respectively. Panel B, high resolution map of *TMEM154* regions targeted for PCR-amplification. PCR amplification primers are indicated with black arrowheads and listed in Table S3. Red lowercase letters above SNP positions are IUPAC/IUBMB ambiguity codes for nucleotides (r = a/g, y = c/t, m = a/c, k = g/t, s = c/g, w = a/t) [17] and indicate 12 sites affected by nonsynonymous substitutions. The red uppercase letters above SNP positions indicate the amino acid polymorphisms encoded at *TMEM154* codons 4, 13, 14, 25, 31, 33, 35, 44, 70, 75, 82 and 102. Black lowercase letters below SNPs indicate nucleotide polymorphisms that resulted in synonymous substitutions. Panel C, the areas of circles for haplotypes 1 to 4 are proportional to the frequencies in the international panel of 75 ISGC sheep. The symbols are as follows: black circles, risk factors; white circle, non-risk factor; grey circles, risk factor status unknown; red circles, haplotypes known in U.S. sheep but not observed in the international panel of 75 ISGC sheep (risk factor status unknown); shaded square, *TMEM154* haplotype predicted to have occurred but unobserved to date. Dashed grey line, haplotypes observed in wild sheep species but not domestic sheep. Haplotypes 13 and 14 were observed in one animal each and their location of origin is indicated.

The fidelity of genetic testing is enhanced by knowing the position and frequency of polymorphisms in the populations to be tested. If not accounted for, nucleotide variation at neighboring sites may cause base-pair mismatching with oligonucleotides used in DNA testing and significantly decrease the genotyping accuracy in some populations. Moreover, characterizing nucleotide variation in many previously untested breeds allows discovery of *TMEM154* missense mutations. For these reasons, the same set of 75 whole genome sequence data was also used to identify novel polymorphisms in the *TMEM154* gene region. An analysis of nucleotide differences among the 75 animals revealed approximately 1500 variant sites in a 61,663 bp region containing *TMEM154*, of which, only 128 had been previously reported [9]. The five wild sheep accounted for 13% of the nucleotide differences observed, however, no heterozygous animals were observed in wild sheep and it was assumed these were mostly species-related nucleotide differences. The positions of these 1,500 SNPs and their minor allele frequencies (MAF) are shown in Figure 2A (blue dots). For comparison, frequency data from 96 rams from 10 U.S. sheep breeds are shown for 128 SNPs (red dots). In regions of *TMEM154* where data are available from both panels of sheep, there was a trend towards more SNPs and higher MAFs in the international panel of 39 breeds compared to the U.S. sheep from 10 breeds.

Four of the previously unreported SNPs were coding mutations located in the predicted signal peptide (A13V) and extracellular domains (E31Q, I74F, and I102T) of *TMEM154*. Variants A13V and I102T were discovered in populations of domestic sheep, whereas E31Q and I74F were found in the wild sheep. The inferred haplotypes for these four putative SNPs were placed in the context of the *TMEM154* median-joining network to provide a framework for their future validation and evaluation of effects on susceptibility to OPPV (Figure 2C).

**Figure 3:**
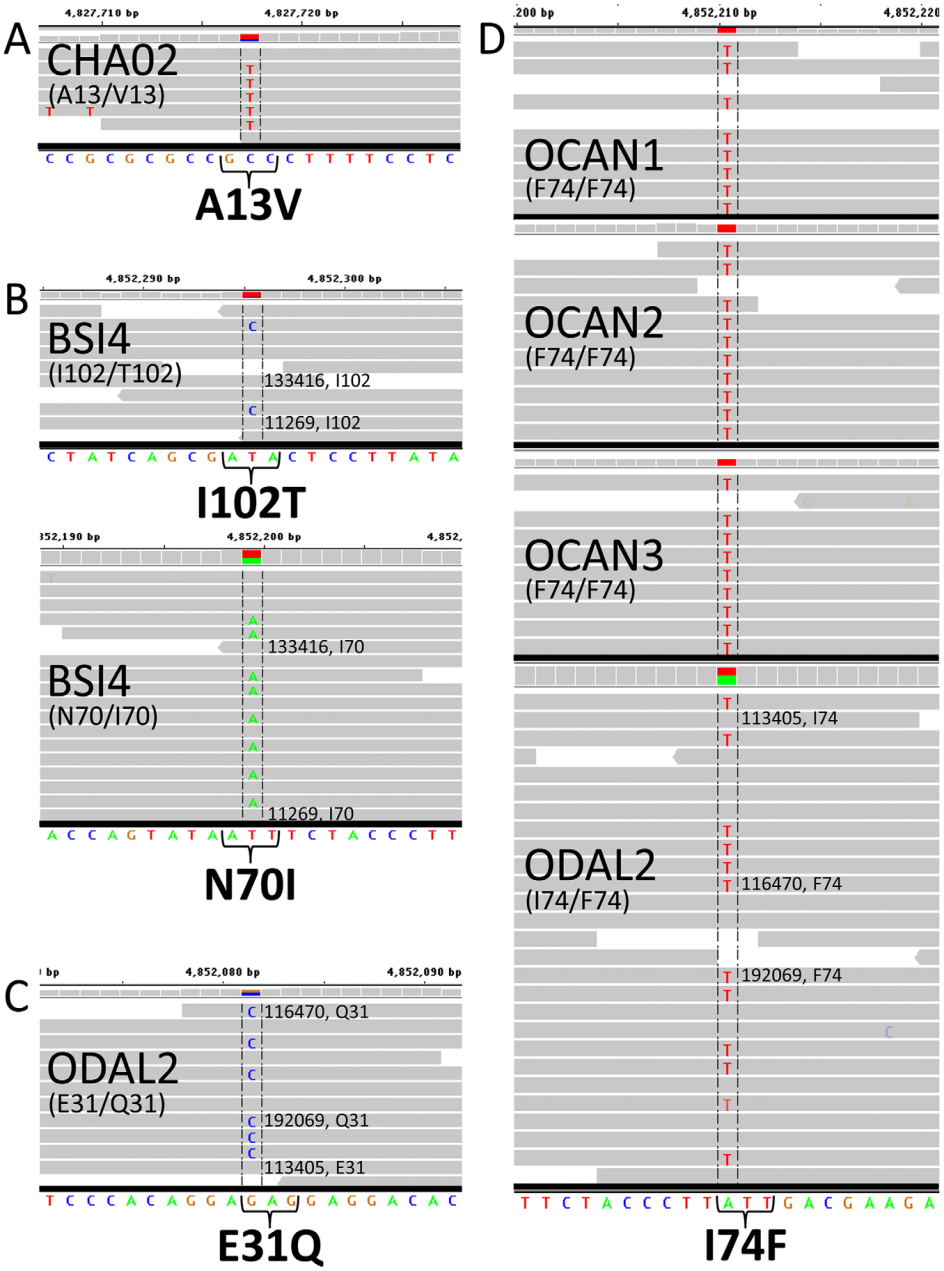
Evidence for *TMEM154* missense mutations in whole genome sequences data from an international panel of 75 sheep. Computer screen images of Integrated Genome Viewer software [18] and showing next generation sequencing reads for animals with previously unreported SNPs affecting the *TMEM154* coding sequence. Numbers shown on the reads indicated the most distal identification number on the read name when viewed in the IGV software. Direct public links to these data are provided: OCAN1 F74/F74, OCAN2 F74/F74, OCAN3F74/F74, ODAL2 I74/F74, CHA02 A13/V13, BSI4 I102/T102, BSI4N70/I70, ODAL2 E31/Q31.

The A13V variant was observed as a heterozygote in a single Changthangi sheep, a local breed in the Changthang area of Leh district of Jammu and Kashmir state (CHA02, Figure 3A). Although the A13V variant was observed in only one animal, five of the seven mapped reads contain the GTC codon for valine. This animal was also homozygous for the *TMEM154* haplotype 9, suggesting that this mutation arose on a haplotype that contained the N33 mutation (Figure 2C, haplotype 13).

The I102T variant was observed as a compound heterozygote in a single Santa Inês sheep, a breed of hair sheep found in Brazil (BSI4, Figure 3B). This animal was also heterozygous for N70I (i.e., haplotypes 2 and 3). Two of nine reads provided evidence for the rare T102 allele and both reads had high quality scores (base Phred quality of 38). Haplotype phase was determined by manual inspection of two other paired-end reads and indicated that the rare T102 allele was on haplotype 3 (Figure 2C, haplotype 14).

The most frequent of the new variants, I74F, was identified in wild sheep (Figure 3D; OCAN1, OCAN2, OCAN3, and ODAL2). The F74 variant was also observed in wild bighorn sheep from Wyoming, USA (n = 10, MAF 0.20, data not shown). A single *Ovis dali* animal from the ISGC set of 75 was also compound heterozygote for I74F and E31Q (ODAL2, Figure 3C). Haplotype phase for these two SNPs was determined by identifying three sets of paired-end reads which each showed that the rare Q31 allele was on the same haplotype as the rare F74 allele (Figure 3C and D). Although F74 and QA31 were only observed in wild sheep, they were placed within the context of the ovine *TMEM154* median-joining network because wild sheep are also susceptible to SRLV. The most parsimonious locations of these haplotypes are shown in Figure 2C (haplotype 15). Whether in domestic sheep or wild sheep, the effects of these previously unreported *TMEM154* missense mutations on susceptibility to OPPV infection are unknown.

### Assigning *TMEM154* Haplotype Pairs (Diplotypes) to Animals

*TMEM154* polymorphisms were scored in all 75 animals at 12 sites: R4A(delta53), A13V, L14H, T25I, E31Q, D33N, E35K, T44M, N70I, I74F, E82Y(delta82), and I102T. Haplotype phase for these 12 sites was unambiguous in 68% of the animals because they had less than two heterozygous sites. Paired haplotypes (i.e., diplotypes) were unambiguously assigned for 100% of the animals by comparing each animal’s genotype to the 78 possible paired combinations of 12 haplotypes (Tables S1 and S2). However, the 20 bp GC–rich region of R4A(delta53) was underrepresented by sequencing reads in 24 of 75 animals. In those animals, the occurrence of M44 was used to infer haplotype 4 by assuming complete linkage disequilibrium with A4(delta53). This assumption is based on the observation that an A4(delta53) allele was always present with an T44 allele, and vice versa, in more than 8,000 U.S. sheep previously genotyped by Sanger sequencing at USMARC [9] (and unpublished data). The most common highly-susceptible haplotypes (2 and 3) were present in 35 of 45 groups in spite of the small sample sizes (Table 1). The average combined frequency of *TMEM154* haplotypes 2 and 3 in the 75 sheep was 0.51±0.08 (Table 2). These results were consistent with those for SNP OAR17_5388531 and confirmed that the highly-susceptible *TMEM154* haplotypes 2 and 3 were widely distributed among the world’s sheep.

**Table 1:** *TMEM154* haplotypes and mutations identified in whole genome sequence data from an international panel of 75 sheep.

Breed or species group	Region of prominence	Animals diplotyped	*TMEM154*haplotypesobserved	Novel missense variants
African White Dorper	South Africa	2	1, 2, 3	–1
Afrikaner, Namaqua	South Africa	1	1, 3	–
Afrikaner, Ronderib	South Africa	2	2, 3	–
Afshari	NW Iran	2	1, 2, 3, 4	–
Awassi	Middle east	1	2, 3	–
Awassi, Turkish	Turkey	2	3	–
Bangladeshi	Bangladesh	2	2, 3	–
Brazilian Creole	Brazil	2	1, 3	–
Castellana	Spain	2	1	–
Changthangi	Jammu and Kashmir	2	3, 9	A13V
Cheviot	England-Scotland	2	1, 2	–
Churra	Spain	2	1	–
Cine Capari	Turkey	1	3, 9	–
Dorset, Poll	USA	1	1	–
Ethiopian Menz	Ethiopia	1	3	–
Finnsheep	Finland	2	1, 2, 3	–
Garole, Banglegdeshi	Bangledesh	1	2	–
Garole, Indian	India	1	2, 3	–
Garut	Indonesia	2	3	–
Gulf Coast native	USA Gulf Coast	2	1, 2	–
Karakas	Turkey	2	1, 2, 3	–
Karya	Turkey	1	2, 4	–
Lacaune, Meat	France	1	1	–
Lacaune, Milk	France	1	1, 4	–
Merino	Spain	3	1, 3	–
Morada Nova	Brazil	2	1, 3	–
Norduz	Turkey	2	1, 3, 4	–
Ojalada	Spain	2	1, 4	–
*Ovis canadensis*	Canada-USA	3	3	I74F
*Ovis dalli*	Canada-USA	2	3	E31Q, I74F
Romney	England	1	1, 2	–
Sakiz	Turkey	2	1, 2, 9	–
Salz	Spain	3	1, 2, 3	–
Santa Inês	Brazil	2	1, 2, 3	I102T
Scottish Blackface	United Kingdom	1	1, 2	–
Sumatra	Sumatra	2	1, 2	–
Swiss Mirror	Switzerland	1	1	–
Swiss White Alpine	Switzerland	4	1, 2, 3	–
Texel	Netherlands	1	2, 3	–
Tibetan, Eastern	Tibet	1	2, 9	–
Tibetan, Northern	Tibet	1	3	–
Valais Blacknose	Switzerland	1	1	–
Welsh Hardy Speckled Face	Wales	1	1	–
Welsh Mountain, Dolgellau	Wales	1	1	–
Welsh Mountain, Tregaron	Wales	1	2	–

1 Not detected.

**Table 2:** Occurrence of *TMEM154* haplotypes in an international panel of 75 sheep.

Haplotype code	Key feature of haplotype	Haplotypes observed^a^	Groups with haplotype^b^
1	K35	56	26
2	I70	34	22
3^c^	E35	42	25
4	A4(delta53)	5	5
6	Y82(delta82)	0	0
9	N33	4	4
10	H14	0	0
11	I25	0	0
12	F74	6	2
13	V13	1	1
14	T102	1	1
15	Q31	1	1

a In 75 sheep.

b Of 45 total groups.

c Ancestral haplotype.

### Reference DNAs for *TMEM154* Testing

The development of genotyping assays for routine high-throughput testing required a set of reference DNAs. Homozygous DNAs were useful as PCR and genotyping controls because they provided uncomplicated results. A minimal set of DNAs was available from animals with the four most common homozygous diplotypes (1,1; 2,2; 3,3; and 4,4) and three rare diplotypes (1,6; 1,9; and 1,10). Together, these seven DNAs provided examples of the eight most common polymorphisms: R4A(delta53), L14H, T25I, D33N, E35K, T44M, N70I, and E82Y(delta82). DNA is not readily available from sheep with the four newly-discovered rare alleles (A13V, E31Q, I74F, and I102T) and thus representative DNA sequences were synthesized and used as controls (Materials and Methods). An empirically determined mixture of synthetic control DNA and reference DNA with *TMEM154* diplotype 1,1 was used to produce a heterozygous MALDI-TOF MS genotype and demonstrated that animals with these rare alleles were detectable in the assay. The combination of the seven reference DNAs, together with four synthetic alleles, provided a minimal set of controls for 12 coding polymorphisms for *TMEM154* for test development and routine assay quality control.

### Essential Regions of *TMEM154* for Genetic Testing

Based on the locations of missense mutations in *TMEM154*, two key regions were targeted for testing: the complete 30-amino acid signal peptide region encoded by exon 1, and residues 31 to 102 of the extracellular domain encoded by exon 2. Three amplicons were designed to encompass the 12 coding polymorphisms on these two exons. One amplicon corresponded to the region of exon 1 containing the R4A(delta53), A13V, L14H, and T25I (Figure 2B). Two other amplicons overlapped each other and corresponded to a region of exon 2 containing E31Q, D33N, E35K, T44M, N70I, I74F, E82Y(delta82), and I102T (Figure 2B).

### MALDI-TOF MS Assay Design and Validation

A three-phase iterative strategy was used to validate the assay development and check concordance of diplotypes derived from MALDI-TOF MS with those derived from Sanger sequencing. In each phase, the samples were blinded, scored, and decoded. Adjustments in assay conditions were made between phases of development. In the first phase, a U.S. panel of 96 rams was genotyped and showed 100% concordance between MALDI-TOF MS and Sanger genotyping. In the second phase, a U.S. panel of 95 tetrad families was used to detect genotyping errors as revealed by non-Mendelian inheritance patterns. One error was detected where the R4 allele was not evident in some animals known to be heterozygous for R4 and A4(delta53) alleles. This apparent allele “dropout” phenomenon was also occasionally observed with R4 alleles scored by Sanger sequencing (data not shown). Although the cause(s) of the R4 allele dropout was unknown, this region of *TMEM154* contains significant secondary structures with melting temperatures near 99 °C and a c/g polymorphism located 10 bp upstream of the R4A(delta53) site. Neither the addition of dimethyl sulfoxide in the PCR cocktail, nor the use of alternative primers in the MALDI-TOF MS extension reaction completely alleviated the occasional R4 allele dropout. Thus, homozygous A4(delta53) genotypes were scored only when they occurred in animals that were also homozygous for M44 in exon 2.

In the last phase of validation, results were obtained for 499 sheep in a single blinded genotyping trial to measure efficiency and accuracy of the genotype assay. Genotyping statistics were calculated for the eight most common polymorphic sites because the remaining four SNPs were monomorphic in these sheep. The call rate for the eight sites was 99.4% for the 499 sheep tested and 96.0% of the animals received a *TMEM154* diplotype assignment (Table 3). Comparing diplotypes from MALDI-TOF MS and Sanger sequencing for 479 animals showed seven discordances (1.46%). Close inspection of raw tracefiles revealed the discordances were due to problems in the Sanger data, including: unamplified alleles, poor quality reads, and errors in manual scoring. These results indicate this MALDI-TOF MS-based test for *TMEM154* provides an accurate alternative to Sanger-based genotyping.

**Table 3:** Call rates and concordance of *TMEM154* genetic testing.

Animal group	Yearsampled	Sheep	Missing SNPgenotypes	SNP callrate^a^ (%)	Missingdiplotypes	Diplotypecall rate	Discordant diplotypes	MALDI-TOFMS errors	Sanger errors
4- to 9-year-old ewes^b^	2003	260	7	99.7	7	0.973	3	0	3
Composite lambs^c^	2011	239	16	99.2	13	0.946	4	0	4
Total	na^d^	499	23	99.4	20	0.960	7	0	7

a Genoptypic data were collected in a single pass for R4A(delta53), H14L, T25I, D33N, E35K, T44M, N70I, and E82Y(delta82).

b This animal group was composed of 160 OPP case-control pairs as previously described [9].

c Breed composition: 1/2 Romanov, 1/4 Rambouillet, 1/8 White Dorper, and 1/8 Katahdin.

d Not applicable.

## Discussion

This report characterizes *TMEM154* in sheep from around the world by estimating the distribution of highly-susceptible haplotypes and identifying novel nucleotide variants. Genotype results from SNP OAR17_5388531 of the ovine SNP50 BeadChip indicated that the two most susceptible alleles (haplotypes 2 and 3) were common and distributed widely. Analysis of next generation sequence for the complete *TMEM154* gene in an international panel of 75 sheep confirmed that the three most common haplotypes described in U.S. sheep populations (haplotypes 1, 2, and 3) are also abundant in groups of sheep sampled from other countries. The most common *TMEM154* haplotype in the international panel of 75 sheep was the less susceptible haplotype 1 encoding lysine at position 35 with a frequency of 0.37. Thus, the opportunity exists in many populations to reduce susceptibility by selectively breeding animals.

The distribution of rare mutant haplotype alleles in populations may provide insight to their history. For example, *TMEM154* haplotypes 6, 10, and 11 (i.e., those with Y82(delta82), H14, and I25 mutations, respectively) were not detected in the international panel of 75 sheep. To date, haplotype 6 has been observed only in Suffolk, and haplotypes 10 and 11 have only been observed in animals with Rambouillet germplasm. In contrast, haplotypes 4 and 9 (i.e., those with A4(delta53)/M44 and N33 mutations, respectively) were observed in breed groups from Turkey, Iran, Spain, France, Tibet, Jammu and Kashmir, suggesting that these haplotypes existed before sheep were brought to the U.S. The occurrence of 12 coding mutations in the predicted signal peptide and extracellular domains of *TMEM154* is remarkable considering that none have been detected in the putative cytosolic domain of *TMEM154* in 309 total sheep from 55 breed groups. However, the biological function of the TMEM154 protein and the impact of these mutations on its function, remain unknown.

The diversity of protein isoforms encoded by the *TMEM154* gene in sheep presents a number of challenges for genetic testing and selective breeding. One challenge is to define a framework for describing the alleles and understanding their action. A frequency-based haplotype numbering system was combined with a rooted median-joining network to put the haplotype-encoded peptide isoforms in a simplified context. Another challenge is to test the haplotypes for their effects on susceptibility to infection while accounting for potential confounders like virus strain, animal exposure, breed type, location, climate, geography, and animal husbandry practices. These factors may potentially overcome the effects of *TMEM154* haplotypes in a given setting. A third challenge is to develop and implement effective *TMEM154* allele management strategies. This will require deploying genetic tests, tracking alleles, and measuring the incidence of infection in a wide variety of settings relevant to commercial sheep production.

The genetic information and assay designs described here were developed so they could be adapted to other platforms and technologies around the world to measure the effects of *TMEM154* haplotypes in local populations. This report also provides the first commercially-available high-throughput genetic test for ovine *TMEM154* haplotypes. The availability of a commercial test is essential for producers to make immediate genetic progress in their respective sheep-improvement programs. As more information becomes available about the effects of *TMEM154* alleles in specific populations and environments, the most appropriate haplotypes can be selected for reducing OPPV susceptibility.

## Materials and Methods

### Ethics Statement

Prior to their implementation, all animal procedures were reviewed and approved by the Care and Use Committee at the United States Department of Agriculture (USDA), Agricultural Research Service, Meat Animal Research Center (USMARC) in Clay Center, Nebraska.

### Animal Samples and Genotypes

The ISGC collected and genotyped 2,819 sheep from 74 breeds as part of a large study into genetic diversity and the impact of selection after domestication [11]. Samples were collected from multiple flocks to minimize relationships within breed. Breeds were collected from the Americas, Africa, Asia, Europe, and the domestication center in present day Iran and Turkey. The geographic origin, breed identity, and number of animals per breed has been previously described [11]. DNA samples were genotyped with the Illumina (San Diego, California USA) ovine SNP50 Beadchip. Genotypes for SNP OAR17_5388531 were available for 2,759 sheep and used for analysis.

The ISGC selected 75 animals for whole genome sequencing to extend its investigation of genetic diversity and selection in the world’s sheep breeds [10]. The majority of the animals (61%) were drawn from the previous study [10] to capture the diversity present across *O. aries*. Additional animals were recruited that either: 1) had previously been used in the construction of genomic resources for the sheep genome [12], 2) carried disease genes, or 3) were wild sheep sampled from the bighorn (*O. canadensis*) and thinhorn (*O. dalli*) populations of North America. Each genome was sequenced and mapped to a read-depth coverage of approximately10-fold with Illumina GAII (unpublished). Prepublication access to the raw sequence data (.bam files) was provided under the Toronto guidelines for data users [13]. These 75 sheep from 39 breed groups and two species groups were used to derive genotypes for the 62 kb genomic region containing *TMEM154.*

The USMARC Sheep Diversity Panel, version 2.4 consists of 96 rams from nine breeds, a composite population, and one Navajo-Churro ram with a rare prion haplotype allele (ARK) as previously described [14]. These rams were part of a set of 96 tetrad families consisting of a ram, a ewe, and twin offspring and used to confirm the haplotype phase of *TMEM154* alleles and to further evaluate the accuracy of genotype scoring. Since the first report of this panel in 2010 [14], family number 47 has been removed because the genotypes from multiple disperse loci for this ram (USMARC Finn no. 200117718) indicate it is not the sire of these offspring. The remaining 95 families were used for testing the accuracy, reproducibility, and segregation of MALDI-TOF MS assays for 12 *TMEM154* polymorphisms affecting 12 codons.

Sheep used for blinded MALDI-TOF MS genotyping trials included 260 USMARC OPP case-control sheep composed of 130 pairs of 4- to 9-year-old ewes [9], and 239 lambs from a 2011 cohort that were 1/2 Rambouillet,1/4 Romanov, 1/8 White Dorper, and 1/8 Katahdin (499 total).

The Wyoming, USA bighorn sheep samples consisted of DNA from 10 wild animals taken from different wildlife management areas across Wyoming prior to 2001.

### Determining *TMEM154* Diplotypes from Whole Genome Sequences

DNA sequence reads from sheep representing 39 *O. aries* breeds plus *O. canadensis and O. dalli* were previously mapped to an ovine reference genome to an average sequencing depth of ten-fold. The UnifiedGenotyper analysis tool from the Genome Analysis Toolkit [15] was used to identify variants, and to genotype the samples at those variant sites. SAMTools viewer [16] was used to extract the subset of the.bam files from each animal’s whole genome sequence files for the locus *TMEM154*. The genomic coordinates used were chromosome 17 positions 4,822,280 to 4,884,987 on the Oar v2.0 map (http://www.livestockgenomics.csiro.au/sheep/). These locus-specific.bam files, their corresponding index files, and genotypes for the variants identified, were loaded into the Intrepid Bioinformatics data management system. Cross reference hyperlinks (/db_xref) were also added to the GenBank file accession HM355886. Diplotypes for the *TMEM154* polypeptide isoforms were constructed by concatenating the genotypes at the 12 polymorphic sites predicted to alter the coding sequence and then matching the concatenated set of genotypes to those listed in Table S1. Because loop structures were not observed in the median-joining network depicted in Figure 2C, the diplotype could be arrived at by only one combination of haplotypes. In rare cases where animals with novel missense SNPs were also heterozygous for nearby missense SNPs, haplotype phase was determined with information from paired-end reads that was accessible in the SAMTools viewer.

### MALDI-TOF MS Genotyping and Synthetic DNA Controls for Rare Alleles

Genotyping was performed at GeneSeek with the Sequenom MassARRAY platform and iPLEX GOLD chemistry according to the manufacturer’s instructions (Sequenom, San Diego, CA USA). Briefly, multiplex assays were designed with commercial software and adjusted manually (see Table S3 for list of oligonucleotides). *TMEM154* exons 1 and 2 were amplified from genomic DNA by PCR and residual oligonucleotides were subsequently dephosphorylated prior to thermocycling in the single-base extension reaction. The oligonucleotide extension products were desalted with size-exclusion resin and transferred to a 384-well chip for MALDI-TOF MS. High-fidelity, long oligonucleotides (Integrated DNA technologies, Coralville, IA USA) with DNA sequences of the 12 minor alleles were synthesized and used as positive control templates in PCR amplification. The lyophilized oligonucleotides were dissolved in 10 mM TrisCl, 1 mM EDTA (pH 8.0) to 25 µM and then empirically diluted to between 1×10^3^ to 1×10^6^ copies per µl before mixing 1∶1 vol/vol with genomic DNA. These samples were subjected to PCR and genotyping as described above. Sequences used for oligonucleotide synthesis included those for *TMEM154* exon 1 (174 bp): tttcagcgggactgacaccgcg[t]gcagcagcatcgc[g]atgccgggg[deleted ‘c’,A4]gcgcgccccccgaggctccgcgcgccg[t,V13]cc[a,H14]tttcctcgccgcggtcctcgcgtcccttccca[t,I25]ccgcccggcgcagggtaagcacccctcggctttccactcccggcgaggaggatgaggaaggctt; *TMEM154*exon 2a (103 bp): gtctcaattttgtatgtgttcccacagga[c,Q31]aggag[a,N33]acaca[g,E35]aactgtcaggagacgtgcccccaggca [t,M44]ggaaggcctggatgaagagtcagaggccctaag; *TMEM154*exon 2b (114 bp): cacacttgcttcagtgaccacagaaccttacatcaccagtataa[t,I70]ttctaccctt[t,F74]ttgacgaagacacagaccagtta [deleted ‘gagttta’,Y82]tattaatggtgttgatcccagtgatttt; and *TMEM154*exon2c (98 bp): actctctctcctgcttctatcagcga[c,T102]actccttataatataccataaaag[g]aaaaggaataaacaaggtaaatattttgcctgttctcatttctaga.

## Supporting Information

Table S1 **List of the 78** ***TMEM154*** **diplotypes possible from 12 known haplotypes.**
(XLSX)

Table S2 **Haplotypes encoding polypeptide isoforms of ovine** ***TMEM154.***
(XLSX)

Table S3 **Oligonucleotide information for** ***TMEM154*** **genetic testing.**
(XLSX)
